# Serum 25-Hydroxyvitamin D Levels Among Boston Trainee Doctors in Winter

**DOI:** 10.3390/nu4030197

**Published:** 2012-03-16

**Authors:** Amanda S. Growdon, Carlos A. Camargo, Sunday Clark, Megan Hannon, Jonathan M. Mansbach

**Affiliations:** 1 Department of Medicine, Children’s Hospital Boston, Harvard Medical School, 300 Longwood AvCe, Boston, MA 02115, USA; Email: Megan.Hannon@childrens.harvard.edu (M.H.); Jonathan.Mansbach@childrens.harvard.edu (J.M.M.); 2 Department of Emergency Medicine, Massachusetts General Hospital, Harvard Medical School, 326 Cambridge St, Suite 410, Boston, MA 02114, USA; Email: ccamargo@partners.org; 3 Department of Medicine, University of Pittsburgh, 200 Meyran Avenue, Suite 300, Pittsburgh, PA 15213, USA; Email: Sunday.Clark@gmail.com

**Keywords:** vitamin D, deficiency, residents, indoor workers

## Abstract

As indoor workers, trainee doctors may be at risk for inadequate vitamin D. All trainee doctors (residents) in a Boston pediatric training program (residency) were invited to complete a survey, and undergo testing for serum 25-hydroxyvitamin D [25(OH)D], PTH, and calcium during a 3-week period in March 2010. We examined the association between resident characteristics and serum 25(OH)D using Chi2 and Kruskal-Wallis test and multivariable linear and logistic regression. Of the 119 residents, 102 (86%) participated. Although the mean serum 25(OH)D level was 67 nmol/L (±26), 25 (25%) had a level <50 nmol/L and 3 (3%) residents had levels <25 nmol/L. In the multivariable model, factors associated with 25(OH)D levels were: female sex (β 12.7, 95% CI 3.6, 21.7), white race (β 21.7, 95% CI 11.7, 31.7), travel to more equatorial latitudes during the past 3 months (β 6.3, 95% CI 2.0, 10.5) and higher daily intake of vitamin D (β 1.1, 95% CI 0.04, 2.1). Although one in four residents in our study had a serum 25(OH)D <50 nmol/L, all of them would have been missed using current Centers for Medicare and Medicaid Services (CMS) screening guidelines. The use of traditional risk factors appears insufficient to identify low vitamin D in indoor workers at northern latitudes.

## 1. Introduction

While vitamin D is known to play an important role in maintaining bone health [1], recent data also suggest many potential non-skeletal health benefits [2]. While the ideal serum level of 25-hydroxyvitamin D [25(OH)D] has yet to be elucidated, 25(OH)D levels of at least 25 nmol/L (to convert to ng/mL divide by 2.496) are required to prevent rickets. The American Academy of Pediatrics [3] (AAP) and the Institute of Medicine [4] (IOM) recommend levels of at least 50 nmol/L to maintain general health, and some experts believe that ideal 25(OH)D levels should be as high as 100 nmol/L [5,6]. Recommendations also vary for daily dietary intake. The U.S. Recommended Dietary Allowance (RDA) for adults was recently increased to 600–800 IU per day depending on age, though others advocate for even higher intake [6]. 

Vitamin D_3_ (cholecalciferol) is produced endogenously when skin is exposed to ultraviolet (UV) B rays, and can also be consumed through dietary intake and nutritional supplements [1]. Because few foods contain vitamin D precursors, exposure to sunlight is the primary determinant of vitamin D status [1]. As a result, the 70% of US workers or nearly 90 million people working indoors [7] may be at risk for low levels of vitamin D. Trainee doctors (also known as “residents” in the US) are one group of workers who spend the majority of their days indoors. At northern latitudes, the risk to indoor workers is further compounded by the lack of sufficient UVB rays in the winter to produce vitamin D in the skin [2,8]. We hypothesized that pediatric residents at northern latitudes would have high rates of serum 25(OH)D <50 nmol/L during winter.

## 2. Experimental Section

### 2.1. Study Participants

This study was approved by the Institutional Review Board. Study participants were enrolled over a three week period in March 2010; all provided informed consent. 

### 2.2. Nutritional and Lifestyle Questionnaires

Participants completed a survey designed to approximate their vitamin D status [9] which included assessments of demographic data, sun exposure, skin sensitivity, travel history, dietary intake of vitamin D, vitamin use over the past 30 days, and medical history. The skin sensitivity scale ranged from 1 (burns easily) to 6 (never burns), and is based on established methods [10]. Data about nutritional supplement use were also collected including: frequency of use, brand name, and dosage of vitamin D and/or calcium. Travel dates and cities travelled to were reported, and latitudes were verified by study authors. Average vitamin D supplement dosage per day in the past 30 days was calculated for each subject based on reports of frequency of use and dose. 

### 2.3. Laboratory Measurements

One blood sample (7.5 mL) was obtained from each subject by study nurses or by study coordinators. Serum 25(OH)D, parathyroid hormone (PTH), and calcium (Ca) were measured. Serum 25(OH)D was tested using a radioimmunoassay on the Nichols Advantage Specialty System (Diasorin, Inc., Stillwater, MN). The intra-assay variation varies from 4.4% to 8.3% and the inter-assay variation from 6.2% to 12.5%. The reference range for intact PTH is 12–88 pg/mL. The intact PTH assay is a chemiluminescent immunoassay using the Access Immunoassay Systems (Beckman Coulter, Brea, CA). The age-appropriate reference-range for calcium is 8.7–10.2 mg/dL.

Each participant was contacted by one of the study authors (AG) to communicate his/her serum 25(OH)D, PTH and Ca levels, and to make recommendations for future intake of vitamin D. Treatment was recommended for 25(OH)D levels <50 nmol/L. Residents with abnormal PTH and/or Ca were referred to their primary care physician for further evaluation.

### 2.4. Data Analysis

All analyses were performed using STATA 11.0 (StataCorp, College Station, TX). Data are presented as proportions [with 95% confidence intervals (CI)], means [with standard deviation (SD)], or medians [with interquartile range (IQR)]. All associations were examined using Chi-squared test, Fisher’s exact test, Student’s *t*-test, and Wilcoxon rank sum test, as appropriate. Variables were evaluated for inclusion in the multivariable models if they were thought to have potential clinical significance or if they were associated with the outcome of interest at *p* < 0.20 in unadjusted analysis. Linear regression was used to evaluate independent predictors of continuous outcomes and logistic regression was used to evaluate independent predictors of dichotomous outcomes. All β coefficients and odds ratios (OR) are presented with 95% CI, and serum 25(OH)D levels were divided into three categories, with low (<50 nmol/L) and high (≥75 nmol/L) being the outcomes of interest. All *p* values are two-tailed, with *p* < 0.05 considered statistically significant. 

## 3. Results

One hundred and nineteen residents were eligible for the study, and 102 (86%) participated. Compared to participants, non-respondents were more likely to be female (66% *vs.* 94%; *p* = 0.02), but did not differ from participants with respect to race or body composition (data not shown). Participants’ demographic information, travel history, nutritional supplement intake and laboratory values are summarized in Table 1. The majority of participants were female (66%), white (74%), and had travelled outside of Massachusetts within 3 months of having their blood drawn (78%). Approximately one-half of the cohort took a dietary supplement in the preceding 30 days, while 25% took either a vitamin D or combination calcium/vitamin D supplement.

The mean serum 25(OH)D level for all study participants was 67 nmol/L (±26). Two participants had low 25(OH)D with abnormally high PTH levels. All participants had normal serum calcium levels. Based on the IOM definition (<50 nmol/L), 25% of participants had inadequate vitamin D. All participants with serum 25(OH)D <50 nmol/L neither had medical conditions known to be associated with vitamin D deficiency nor did they take medications known to interfere with absorption or metabolism of vitamin D. Furthermore, 11 of these 25 residents were not dark skinned or obese, both established risk factors for low vitamin D [11,12].

**Table 1:** Overview of Pediatric Resident Survey.

Characteristic	*n* = 102
**Demographics & Level of Training**	
Age, years (mean ± SD)	29.6 ± 2.5
Age (%)	
<30 years	53 (52%)
≥30 years	49 (48%)
Sex (%)	
Female	67 (66%)
Male	35 (34%)
Ethnicity (%)	
Hispanic or Latino	2 (2%)
Not Hispanic or Latino	100 (98%)
Race (%) (*n*= 1 missing)	
White	75 (74%)
Black or African American	3 (3%)
Asian	23 (23%)
Resident level of training (%)	
Intern	34 (33%)
Junior	36 (35%)
Senior	32 (31%)
**Clinical Factors**	
Body mass index, kg/m^2^ (mean ± SD)	22.7 ± 2.9
BMI (%)	
<25 kg/m^2^	79 (77%)
25–29 kg/m^2^	22 (22%)
≥30 kg/m^2^	1 (1%)
Skin Sensitivity Score (%) *	
1	6 (6%)
2	19 (19%)
3	42 (41%)
4	17 (17%)
5	16 (16%)
6	2 (2%)
Have any of the following doctor-diagnosed illnesses (%) ^†^	4 (4%)
**Travel History**	
Traveled outside of Massachusetts in past 3 months (%)	80 (78%)
If yes, lowest latitude traveled to in past 3 months (mean ± SD)	32 ± 10
If yes, applied sunscreen of SPF 15 or greater to face or body at least once per day during travels (%)	34 (43%)
**Nutritional Supplement Use During the Past 30 Days**	
Taken any vitamin or supplement in the past 30 days (%)	49 (48%)
Taken multivitamin in past 30 days (%)	44 (43%)
In past 30 days, number of days vitamin taken (%)	
0–14 days	28 (64%)
15+ days	16 (36%)
Taken vitamin D or dual calcium/vitamin D supplement in past 30 days (%)	25 (25%)
Taken vitamin D supplement in past 30 days (%)	11 (11%)
In past 30 days, number of days vitamin taken (%) (*n* = 1 missing)	
0–14 days	6 (60%)
15+ days	4 (40%)
Taken dual calcium/vitamin D supplement in past 30 days (%)	16 (16%)
In past 30 days, number of days vitamin taken (%)	
0–14 days	11 (69%)
15+ days	5 (31%)
Vitamin D supplement dosage in past 30 days	
None	77 (75%)
200–600 IU	12 (12%)
≥800 IU	13 (13%)
Taken cod liver oil in past 30 days (%)	1 (1%)
**Laboratory Values**	
Serum 25 (OH) D, nmol/L (mean ± SD)	67 ± 26
Serum 25(OH)D levels (%)	
<25 nmol/L	3 (3%)
25–49 nmol/L	22 (22%)
50–74 nmol/L	45 (44%)
75–99 nmol/L	22 (22%)
≥100 nmol/L	10 (10%)
PTH, pg/mL (median [IQR])	32.3 (24.9–42.5)
Calcium, mg/dL (mean ± SD)	9.4 ± 0.3

Abbreviations: 25(OH)D: 25-hydroxyvitamin D; SD: Standard deviation; BMI: Body mass index; SPF: Sun protection factor; PTH: Parathyroid hormone; IQR: Interquartile range. * See Methods section for details. ^†^ Doctor-diagnosed illnesses include malabsorptive diseases including inflammatory bowel disease, celiac disease, gastric bypass surgery, liver failure, nephritic syndrome or chronic kidney disease, granulomatous disorders such as sarcoid or TB, heritable rickets or untreated hyperthyroidism.

As shown in Table 2, white race, multivitamin use and vitamin D supplement use in the past 30 days were significantly associated with higher 25(OH)D levels. Female sex and travel to more equatorial latitudes were of borderline statistical significance. Dietary intake of vitamin D-fortified milk, vitamin D-fortified juice, salmon and canned tuna were not associated with higher serum 25(OH)D levels (data not shown), nor was taking a combination calcium/vitamin D supplement. As anticipated, PTH values were lower in participants with higher serum 25(OH)D levels. 

**Table 2:** Resident Characteristics and Dietary Patterns According to Serum 25(OH)D Level *.

Characteristic	<50 nmol/L *n* = 25 (25%)	50–74 nmol/L *n* = 45 (44%)	≥75 nmol/L *n* = 32 (31%)	*p* value
**Demographics & Level of Training**				
Age, years (mean ± SD)	30.0 ± 1.6	29.3 ± 2.9	29.7 ± 2.5	0.52
Sex (%)				0.06
Female	18	45	37	
Male	37	43	20	
Ethnicity (%)				0.31
Hispanic or Latino	50	0	50	
Not Hispanic or Latino	24	45	31	
Race (%) (*n* = 1 missing)				0.01
White	16	47	37	
Black or African American	33	67	0	
Asian	48	35	17	
**Clinical Factors**				
Body mass index, kg/m^2^ (mean ± SD)	23.8 ± 3.9	22.3 ± 2.4	22.4 ± 2.4	0.09
Skin Sensitivity Score (%) ^†^				0.13
1–2	16	48	36	
3–4	20	46	34	
5–6	50	33	17	
**Travel History**				
Traveled outside of Massachusetts in past 3 months (%)	25	41	34	0.48
If yes, lowest latitude traveled to in past 3 months (mean ± SD)	35 ± 9	33 ± 11	29 ± 11	0.08
**Nutritional Supplement Use During the 30 Days**				
Taken any vitamin or supplement in the past 30 days (%)	14	49	37	0.07
Taken multivitamin in past 30 days (%)	9	52	39	0.005
In past 30 days, number of days vitamin taken (%) (*n* = 1 missing)				0.90
0–14 days	11	54	36	
15+ days	6	50	44	
Taken vitamin D or dual calcium/vitamin D supplement in past 30 days (%)	20	32	48	0.13
Taken vitamin D supplement in past 30 days (%)	0	36	64	0.03
In past 30 days, number of days vitamin taken (%) (*n* = 1 missing)				1.00
0–14 days	--	33	67	
15+ days	--	25	75	
**Nutritional Supplement Use During the 30 Days**				
Taken dual calcium/vitamin D supplement in past 30 days (%)	31	31	38	0.57
In past 30 days, number of days vitamin taken (%)				0.06
0–14 days	45	36	18	
15+ days	0	20	80	
Vitamin D supplement dosage in past 30 days				0.33
None	26	48	26	
200–600 IU	25	33	42	
≥800 IU	15	31	54	
Taken cod liver oil in past 30 days (%)	0	100	0	1.00
**Laboratory Values**				
Serum 25 (OH) D, nmol/L	37.1 ± 8.9	62.2 ± 6.6	96.6 ± 19.9	--
PTH, pg/mL (median [IQR])	47.3 (33.3–55.8)	32.0 (26.4–37.7)	25.9 (21.3–33.5)	<0.001
Calcium, mg/dL (mean ± SD)	9.4 ± 0.3	9.4 ± 0.3	9.4 ± 0.3	0.90

Abbreviations: 25(OH)D: 25-hydroxyvitamin D; SD: Standard deviation; BMI: Body mass index; PTH: Parathyroid hormone; IQR: Interquartile range. * Percentages are for each row. ^†^ See Methods section for details. ^‡^ Doctor-diagnosed illnesses include malabsorptive diseases including inflammatory bowel disease, celiac disease, gastric bypass surgery, liver failure, nephritic syndrome or chronic kidney disease, granulomatous disorders such as sarcoid or TB, heritable rickets or untreated hyperthyroidism.

Multivariable analysis identified several factors independently associated with serum 25(OH)D levels (Table 3). When serum 25(OH)D levels were analyzed as a continuous variable the factors associated with 25(OH)D levels were: female sex, white race, travel to more equatorial latitudes and higher total daily intake of vitamin D from supplements. The results were consistent when looking at low (<50 nmol/L) and high (≥75 nmol/L) serum 25(OH)D groups. 

**Table 3:** Predictors of 25(OH)D Levels.

Characteristic	Predictors of 25(OH)D Levels (nmol/L)	Predictors of Low 25(OH)D Levels (<50 nmol/L)	Predictors of High 25(OH)D Levels (≥75 nmol/L)
	β (95% CI)	OR (95% CI)	OR (95% CI)
Female	12.7 (3.6, 21.7) *p* = 0.007	0.3 (0.1–0.7) *p* = 0.01	3.2 (1.1–9.4) *p* = 0.04
White race	21.7 (11.7, 31.7) *p* < 0.001	0.2 (0.1–0.6) *p* = 0.002	3.5 (1.0–12.4) *p* = 0.051
If traveled in past 3 months, lowest latitude traveled to (per ↓10° latitude) *	6.3 (2.0, 10.5) *p* = 0.005	0.6 (0.3–1.1) *p* = 0.10	1.8 (1.1–2.8) *p* = 0.01
Vitamin D supplement dosage in past 30 days (per ↑100 IU)	1.1 (0.04, 2.1) *p* = 0.04	0.9 (0.8–1.1) *p* = 0.49	1.1 (1.0–1.3) *p* = 0.04

Abbreviations: 25(OH)D: 25-hydroxyvitamin D; β: β-coefficient; OR: Odds ratio; * Individuals who did not travel during the past 3 months were assigned the latitude of Boston (42°).

## 4. Discussion

One in four residents in this sample had inadequate vitamin D (defined as 25(OH)D <50 nmol/L) even though this cohort is markedly enriched for subjects with demographic characteristics traditionally associated with higher serum 25(OH)D levels including high educational level [13], younger age [1], white race [14], low BMI [15] and good health [16]. Given these sample characteristics, other workers who spend their daylight hours indoors and reside at higher latitudes may be at similar risk. Therefore, performing vitamin D screening based on traditional risk factors appears insufficient to identify indoor workers living at northern latitudes who have inadequate vitamin D. In those workers with vitamin D <50 nmol/L, most experts recommend treatment with a high-dose vitamin D supplement (e.g., 50,000 IU per week for 6–8 weeks) and then repeat 25(OH)D testing [11,17]. 

In our sample, female sex was an independent factor associated with higher serum 25(OH)D levels. This finding differs from previous population-based studies that have noted higher serum 25(OH)D levels in men compared to women [18]. Nationwide, men are more likely than women to work outdoors and may therefore have more sun exposure and higher serum 25(OH)D levels. In contrast, all residents follow a similar work schedule that requires them to be indoors for the majority of daylight hours. Therefore, there may be increased importance of vitamin D supplementation in this population. Similar to national trends [19], women in our study were more likely to take nutritional supplements than men which may account for some of the observed gender difference.

The type of nutritional supplement taken was also associated with serum 25(OH)D levels. Multivitamin and vitamin D supplement use were associated with higher serum 25(OH)D levels, but use of combined calcium/vitamin D supplements were not. The lack of association with combined supplements may be due to the small numbers of residents taking this type of supplement, but may also be due to the lower vitamin D dose typically contained in the combined supplements (e.g., approximately 500 IU daily). Among residents taking either a vitamin D only or combination calcium/vitamin D supplement, an increasing daily dose of vitamin D was associated with higher levels of serum 25(OH)D, suggesting that the difference was dose-related. 

As screening for low vitamin D has become more common, some have advocated against wide-spread testing, and instead are recommending universal supplementation in accordance with IOM guidelines [4]. Indeed, per current Endocrine Society guidelines, it is recommended that primary care physicians reserve serum 25(OH)D testing for patients in defined higher risk categories [11]. Furthermore, the Centers for Medicare and Medicaid Services (CMS) have mandated that “vitamin D testing may not be used for routine screening” and can only be performed on patients with medical conditions known to be associated with inadequate vitamin D levels. These recommendations are of some concern because even in our relatively homogeneous population with few identified risk factors and good overall health, serum 25(OH)D levels ranged widely. Of the 25% of residents in our study with serum 25(OH)D <50 nmol/L, none had medical conditions known to be associated with low levels of vitamin D. Therefore, several residents with inadequate serum 25(OH)D would not have been identified if testing were performed only on those participants with known demographic and lifestyle risk factors. And for those with 25(OH)D levels <50 nmol/L, the recommendation would be to provide treatment doses of vitamin D (e.g., 50,000 IU per week for 6–8 weeks) and then to begin standard daily dose of vitamin D [11,17]. Given that one in four residents had levels <50 nmol/L, that the amount of time residents spend indoors may not be that different from other employees, and the established health benefits of treating inadequate levels of 25(OH)D [4], we suggest consideration of routine testing of serum 25(OH)D in indoor workers at northern latitudes during winter.

Our study was limited by its relatively small sample size, though we were able to enroll 86% of eligible participants. Moreover, our population was relatively homogeneous, with few residents from higher risk groups including racial and ethnic minority groups, those with high BMI or medical illnesses. However, given that these personal characteristics are generally associated with lower levels of serum 25(OH)D, our results may represent a best case scenario for workers living in northern latitudes who spend their days indoors. Importantly, this study was conducted in the northern US, which probably limits generalizability of the results to more equatorial latitudes where great UVB exposure still can stimulate endogenous vitamin D production. On the other hand, recent studies are finding that low levels of vitamin D are found in a variety of sunny locations worldwide as people increasingly adopt indoor lifestyles [20,21,22,23]. 

## 5. Conclusions

These data suggest that trainee doctors in northern latitudes are at high risk of inadequate vitamin D levels during winter. Other groups of indoor workers at similar latitudes may also be at risk, particularly given the unusually favorable demographic characteristics of our cohort. Based on traditionally identified risk factors, only a fraction of all residents with low serum 25(OH)D levels would have been categorized as being at “high risk.” Moreover, in those with low serum 25(OH)D, routine supplementation may be inadequate to replete body stores. Therefore, while some expert panels [4,11] and insurance companies [24] have advocated against routine screening, it may be appropriate to consider winter screening for indoor workers residing at northern latitudes. Residency programs (and other professions where workers spend the majority of their time indoors) may want to consider educating their residents on the physical effects of prolonged hours indoors, including the risks of inadequate vitamin D.

## Implications

Indoor workers in northern latitudes may be at risk for vitamin D inadequacy during winter, even without other identifiable risk factors. Screening should be considered in this population of workers.
